# Dramatic Responses to Low-Dose Pramipexole in Painful Legs and Moving Toes Syndrome

**DOI:** 10.7759/cureus.34763

**Published:** 2023-02-08

**Authors:** Gakusuke Umezawa, Takafumi Hasegawa, Kensuke Ikeda, Genichi Saito, Masashi Aoki

**Affiliations:** 1 Department of Neurology, Tohoku University School of Medicine, Sendai, JPN; 2 Department of Neurology, Jinmei Saito Hospital, Ishinomaki, JPN

**Keywords:** plmts, pramipexole, dopamine agonist, involuntary movement, moving toes, painful legs

## Abstract

Painful legs and moving toes syndrome (PLMTS) is a rare movement disorder characterized by spontaneous abnormal, involuntary toe movements and unilateral or bilateral lower extremity pain that predominantly affects women in middle age or later. The background etiology of PLMTS includes peripheral neuropathy, a history of trauma, and nerve root damage, but the cause of the disease is often undetermined. The pain usually occurs first and is often more distressing to the patient than abnormal toe movement. Spontaneous resolution is rare, and symptomatic therapies include the oral administration of anticonvulsants, antidepressants, and various pain relievers, as well as other therapeutic interventions, including botulinum toxin injection and epidural block, but their effectiveness is uncertain. We report a case of PMLTS in which low doses of pramipexole, a non-ergot dopamine agonist, dramatically improved both abnormal toe movement and leg pain, which are documented by videography.

## Introduction

Painful legs and moving toes syndrome (PLMTS), first described by Spillane et al. in 1971, is a rare clinical condition characterized by involuntary toe movements accompanied by discomfort and pain sensation in the legs [1]. The nature of the abnormal toe movements varies, and patients describe them as flexion-extension, fanning, or circular movements. Many patients are affected more by persistent, severe pain than involuntary movements [1,2]. Peripheral neuropathy, history of trauma, and radiculopathy have been reported as the causative conditions of PLMTS, but the etiology cannot be identified in 40% of cases [2-4]. Here, we report a case of idiopathic PLMTS in which low-dose dopamine agonist, pramipexole, showed a significant effect on both leg pain and abnormal toe movements.

## Case presentation

A woman in her seventies developed persistent burning pain in her right toes and ankle five years ago, accompanied by constant, involuntary twitching movements in the same areas. Her past medical history included hypertension, dyslipidemia, and depression. No history of taking antidepressants or antipsychotics in the past few years. There was no family history of consanguineous marriage or neuromuscular disease. The patient was referred to our hospital as a local hospital failed to reach a diagnosis. Neurological examination on admission revealed persistent involuntary movement comprising abduction-adduction and twisting movements of the right toes, as well as the flexion-extension movement of the right ankle, accompanied by pain. No parkinsonism was observed. These abnormal movements were less noticeable during walking (Video 1). In addition to being accompanied by sustained severe leg pain, the abnormal movements, in this case, were distinct from the symptom of restless legs syndrome (RLS), i.e., they appeared involuntary, were not accompanied by an irresistible urge to move, and did not fluctuate within the day, such as being more likely to occur in the evening or at night [5]. An extensive survey, including routine blood and urine tests, biochemical analysis of cerebrospinal fluid, magnetic resonance imaging of the brain and spine, and nerve conduction studies, was unremarkable. Based on the characteristic nature of the involuntary movements with excruciating pain and the lack of background etiology, a diagnosis of idiopathic PLMTS was made. The first therapeutic intervention of clonazepam (0.5 mg/day) failed; however, after taking a low-dose non-ergot dopamine agonist, pramipexole (0.25 mg before bedtime), her involuntary movement disappeared quickly and completely and the pain greatly relieved (Video 2). At six months after discharge, the treatment was still effective with no adverse effects.

**Video 1:** Involuntary movements of the right toes and ankle joint before treatment. The patient shows involuntary movement consisting of abduction-adduction and twisting movements of the right toes, as well as the flexion-extension movement of the right ankle, accompanied by pain. These abnormal movements are not noticeable during walking.

**Video 2:** Changes in motor symptoms of the toes and ankle joints after two days of oral pramipexole. Oral administration of pramipexole (0.25 mg before bedtime) markedly alleviates involuntary movements, and the pain is greatly relieved.

## Discussion

Although the pathophysiology of PLMT remains unclear, there are a certain number of cases in which peripheral neuropathy or nerve root involvement is thought to be a background etiology [2]. Nathan hypothesized that impulses generated by lesions in the peripheral nerves, dorsal root ganglia, and cauda equina may be responsible for transmitting pain to the central nervous system (CNS), while spinal interneurons may excite anterior horn cells, resulting in involuntary movements [6]. In addition, several reports claimed that sympathetic blockade was effective in improving symptoms [1,4], suggesting the involvement of the sympathetic nervous system in the pathogenesis of PLMTS. Although very rare, there have been reports of *painless* legs and moving toes associated with Wilson's disease and parasagittal meningioma [7,8], suggesting that functional abnormalities in the CNS may also be involved in the pathogenesis of PLMTS.

Treatment of PLMTS is often challenging. Oral medications such as GABAergic agents, benzodiazepines, dopaminergic agents, antiepileptic agents, and other therapeutic interventions (e.g., botulinum toxin injection, epidural block, and local anesthetic nerve block) have been tried, with limited success [4]. The lack of reliable treatment, combined with a lack of understanding by those around them, leads many patients to become depressed. Because the underlying etiology of PLMTS varies from case to case, it is easy to imagine that treatment efficacy is inconsistent. In general, the degree of involuntary movements and pain often correlate with each other in PLMTS, but in some cases, there is a discrepancy in treatment efficacy [2,9]. These variations in treatment responsiveness also raise the various pathological mechanisms.

From a clinical perspective, PLMTS shares some characteristics with RLS in that both cause abnormal motion and pain in the lower extremities. Our patient did not respond to clonazepam but showed marked improvement with pramipexole, suggesting the involvement of the dopaminergic system in the pathogenesis. In RLS, many patients show favorable responses to dopamine agonists [10], and intriguingly, there are a small number of cases of PLMTS, in which non-ergot dopamine agonists such as pramipexole, rotigotine, and ropinirole were effective [2,11,12]. Pramipexole is a dopamine D2 and D3 receptor agonist, which is widely used for the treatment of motor symptoms in Parkinson’s disease and RLS [10]. The pharmacological mechanism by which pramipexole alleviates the symptoms of PLMTS remains unknown; however, in a rodent model of RLS caused by the lesioning of diencephalic-spinal A11 dopamine neuron, pramipexole administration does not alter dopamine content in the spinal cord but reduces dopamine D3 receptor density and enhances D1 receptor affinity [13,14]. These results raise a possibility that pramipexole might reduce PLMTS symptoms by modulating dopamine receptor function in the spinal cord. Despite various hypotheses described earlier, many of the pathomechanisms of PLMTS remain to be elucidated and require further study.

## Conclusions

We describe an adult case of PMLTS in which low doses of pramipexole markedly resolved both abnormal toe movement and leg pain. PLMTS has a significant impact on the patient's quality of life, but its presence is not well known to healthcare professionals, often leading to delays in diagnosis and treatment. The phenomenology of involuntary movement is often unfamiliar to all but experienced neurologists. We believe that this case report will be helpful in the early diagnosis and treatment selection of PLMTS.
